# Production and Characterization of Austenitic Stainless Steel Cast Parts Reinforced with WC Particles Fabricated by Ex Situ Technique

**DOI:** 10.3390/ma14247855

**Published:** 2021-12-18

**Authors:** Aida B. Moreira, Laura M. M. Ribeiro, Pedro Lacerda, Ana M. P. Pinto, Manuel F. Vieira

**Affiliations:** 1 Department of Metallurgical and Materials Engineering, University of Porto, R. Dr. Roberto Frias, 4200-465 Porto, Portugal; up201108098@fe.up.pt (A.B.M.); lribeiro@fe.up.pt (L.M.M.R.); 2 LAETA/INEGI—Institute of Science and Innovation in Mechanical and Industrial Engineering, R. Dr. Roberto Frias, 4200-465 Porto, Portugal; 3 FERESPE, Fundição de Ferro e Aço Lda., R. Basileia, 4760-485 Vila Nova de Famalicão, Portugal; pedrolacerda@ferespe.pt; 4 CMEMS—Center for MicroElectroMechanics Systems, Department of Mechanical Engineering, University of Minho, 4800-058 Guimarães, Portugal; anapinto@dem.uminho.pt

**Keywords:** austenitic stainless steel, ex situ technique, local reinforcement, metal matrix composite, microstructural characterization, tungsten carbide

## Abstract

In this work, austenitic stainless steel specimens were locally reinforced with WC particles. The reinforcements were fabricated via an ex situ technique based on powder technology. Mixtures of WC, Fe, and M0101 binder were cold-pressed to obtain powder compacts. After debinding and sintering, the porous WC–Fe inserts were fixed in a mold cavity, where they reacted with liquid metal. Microstructural analysis was conducted for characterization of the phases constituting the produced reinforcement zone and the bonding interface. The results revealed that the reinforcement is a graded material with compositional and microstructural gradients throughout its thickness. The zone nearest to the surface has a ferrous matrix with homogeneously distributed WC particles and (Fe,W,Cr)_6_C and (Fe,W,Cr)_3_C carbides, formed from the liquid metal reaction with the insert. This precipitation leads to austenite destabilization, which transforms into martensite during cooling. A vast dissolution of the WC particles occurred in the inner zones, resulting in more intense carbides formation. Cr-rich carbides ((Fe,Cr,W)_7_C_3_, and (Fe,Cr,W)_23_C_6_) formed in the interdendritic regions of austenite; this zone is characterized by coarse dendrites of austenite and a multi-phase interdendritic network composed of carbides. An interface free of discontinuities and porosities indicates good bonding of the reinforcement zone to stainless steel.

## 1. Introduction

Austenitic stainless steels are the largest family of stainless steels in terms of number of alloys and applications. These steels are widely used due to their high corrosion resistance in aqueous media at room temperature and in applications with hot gases and liquids at high temperatures, such as sulfuric acid environments with temperatures up to 540 °C [1]. The excellent corrosion resistance combined with high mechanical strength, toughness, conformability, and weldability have promoted their extensive use in components for maritime, petrochemical, chemical, nuclear, biomedical, pharmaceutical, and food processing industries [1,2,3,4].

Despite the broad range of industrial applications, austenitic stainless steels show poor wear resistance that limits their use in components for industrial applications that demand both high corrosion and wear resistance [5,6,7]. Several techniques can be applied for surface modification to improve the wear resistance of the outer surface of stainless steel parts using hard coating materials and composites such as physical vapor deposition and chemical vapor deposition [8,9,10], laser cladding or laser surface alloying [11,12,13], plasma cladding [14,15], arc welding [16,17], and brazing [18,19,20]. However, apart from specific equipment with high associated costs, these procedures represent another step of the manufacturing process, increasing the manufacturing time. Thus, the development of metal matrix composites (MMCs) locally reinforced with carbides offers a way to reinforce stainless steel cast components, enhancing the surface wear resistance without compromising the corrosion and mechanical properties [2,21,22,23]. The great advantage of this method is that it makes it possible to produce components of complex geometry with high corrosion and wear performance [24,25,26].

The production of WC-reinforced cast steel components has been studied since the first decade of the century. The majority of the studies have been focused on pressure-driven infiltration processing [27,28] and lost model technique [29,30,31]. There are also investigations concerning pressureless infiltration [32] and centrifugal casting processing [33,34]. However, to our best knowledge, this is the first study focused on the production of austenitic stainless steel parts locally reinforced with WC particles by pressureless infiltration.

Various chromium-rich phases such as carbides, sigma (σ), and chi (χ) may precipitate in the temperature range of 650–1010 °C [1,35] in the austenitic stainless steels. The precipitation of such phases causes the Cr reduction of the matrix, leading to sensitization and decreased corrosion resistance of the steel. Therefore, the steel has to be heat-treated in the temperature range of 1040–1205 °C to ensure the solution of rich Cr phases [1,36,37], followed by water quenching. A major challenge of the production of reinforced stainless steel cast components is to understand the effect of the solution heat treatment on the microstructure of the reinforcement zone and the bonding interface, which should resist residual stresses that are induced due to the mismatch of the coefficients of thermal expansion between the base metal and the reinforcement zone. In this way, this research aims to investigate the manufacture of austenitic stainless steel cast specimens reinforced with WC particles fabricated by an ex situ technique. This work provides a detailed characterization of the phases formed in the composite zone, which is a key issue for understanding the bonding mechanism, and, consequently, achieving high-quality reinforced components. Therefore, sintered WC–Fe inserts with high porosity for improving the liquid metal infiltration were placed in the mold cavity before pouring the stainless steel to obtain the reinforced stainless steel specimens.

## 2. Materials and Methods

**Figure 1:**
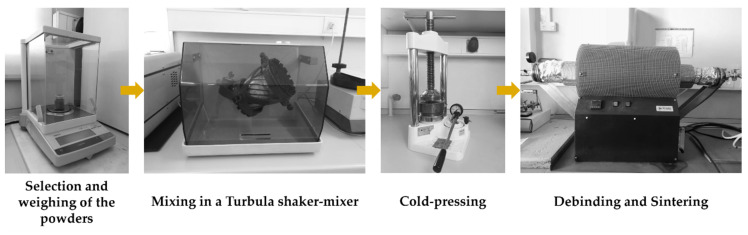
Fabrication steps for the Fe–WC inserts production.

**Figure 2:**
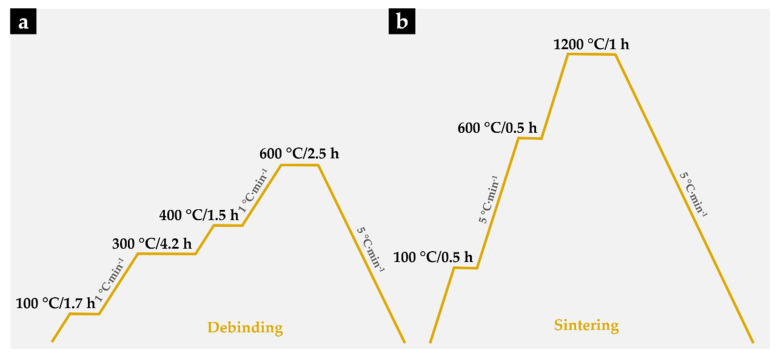
Debinding and sintering thermal cycles applied to Fe–WC compacts. (**a**) The thermal cycle carried out during debinding; (**b**) The applied thermal cycle with heating and cooling rates of 5 °C∙min^−1^.

**Figure 3:**
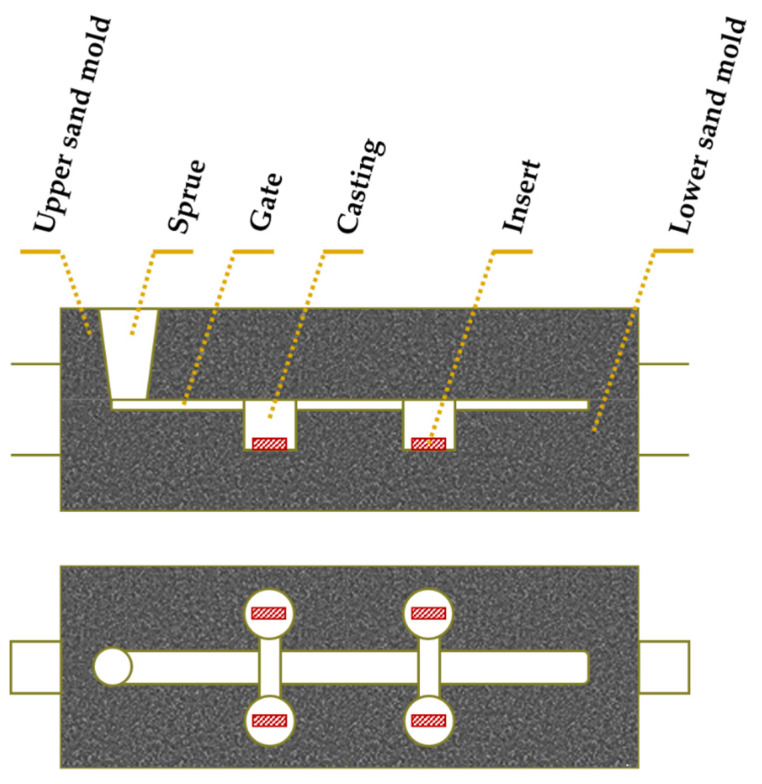
Scheme of the mold with the Fe–WC inserts (side and top views) (adapted from [40]).

**Figure 4:**
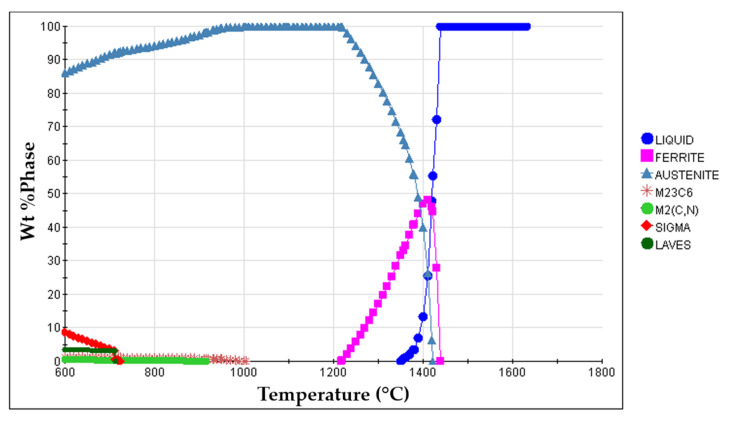
Equilibrium phase diagram of the GX5CrNiMo19-11-2 alloy (ISO 4991:2015) calculated with JMatPro^TM^ v.11.2 software.

**Figure 5:**
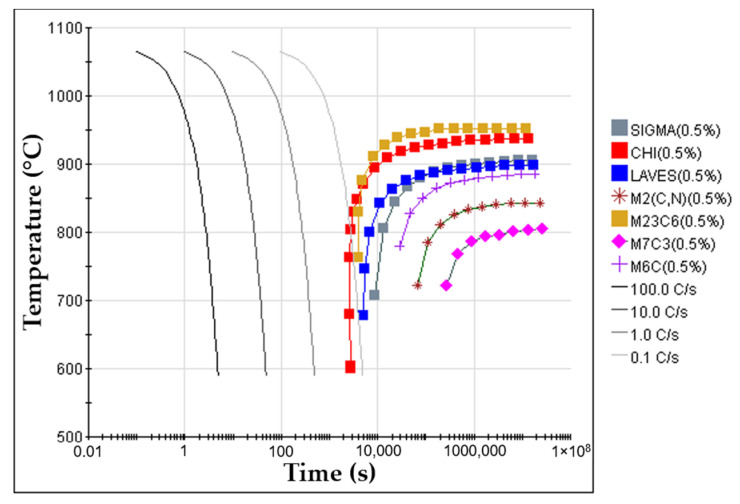
Continuous cooling transformation diagram (CCT) of GX5CrNiMo19-11-2 alloy (ISO 4991:2015) for an austenitizing temperature of 1075 °C, calculated with JMatPro^TM^ v.11.2 software.

The reinforced specimens were produced by casting. The fabrication method has been given in our previous papers [38,39], but with the difference that porous Fe–WC inserts were prepared using the powder technology (see Figure 1). The production steps of the reinforced specimens were as follows:

1. Selection and characterization of the initial powders. Commercial powders of WC (99.0 wt % purity) and Fe (99.0 wt % purity), from Alfa Aesar, Thermo Fisher (Kandel, Germany) GmbH, and a commercial binder material (designated as Full-mould MIM binder M0101) from Atect Corporation (Shiga, Japan), composed of Polyolefin-modified polyoxymethylene (≥60.0 wt %), paraffin wax (≤20.0 wt %), and ester wax (≥20.0 wt %), were chosen. The powders were characterized by scanning electron microscopy (SEM), using a FEI QUANTA 400 FEG (FEI Company, Hillsboro, OR, USA) with an energy-dispersive detector (EDS) and dynamic light scattering (DLS, Laser Coulter LS230 granulometer, Beckman Coulter, Inc., Brea, CA, USA) techniques.
2. Mixing and homogenization of the powders. Powders of Fe (60 vol %) and WC (40 vol %), and the binder were mixed, in a volume fraction of 70:30, in a Turbula shaker-mixer (Willy A. Bachofen AG, Muttenz, Switzerland) for 7 h.
3. Cold-pressing of the whole mixture. The mixture was uniaxially cold-pressed at 230 MPa in a metallic mold to produce green compacts (31 mm × 12 mm × 9 mm).
4. Debinding. At this stage of the process, the binder was removed to produce porous brown compacts. This process was carried out on a horizontal resistance furnace using an argon–hydrogen (5 vol % H_2_) atmosphere. The thermal cycle carried out during this stage is shown in Figure 2a.
5. Sintering the brown compacts. After the debinding, the compacts were sintered at 1200 °C for one hour in a horizontal resistance furnace equipped with a high-vacuum turbo molecular pump (≤5 × 10^−3^ Pa). The applied thermal cycle with heating and cooling rates of 5 °C∙min^−1^ is depicted in Figure 2b.

6. Placement of porous sintered Fe–WC inserts in the mold cavity. Before casting the liquid metal, the inserts were positioned in the desired location of the mold cavity (see Figure 3).
7. Melting and casting. The melt was prepared in a medium frequency induction furnace, with a capacity of up to 1000 kg, and poured at 1620 °C into the mold cavity. The nominal chemical composition of the liquid metal, in accordance with the ISO 4991:2015 standard, shown in Table 1, was analyzed by optical emission spectrometry (MAXx LMM05, Spectro, Germany).
8. Solution heat treatment. The solution heat treatment is a required step to dissolve brittle and Cr-rich phases that may form during the cooling of the steel [1,36,37]. These phases are sigma (σ), Laves, M_2_N, M_2_C, and M_23_C_6_, predicted from the equilibrium phase diagram calculated with JMatPro^TM^ v.11.2 and shown in Figure 4. From the continuous cooling transformation diagram (CCT), also calculated with JMatPro^TM^ v.11.2, a fast-cooling rate (approximately 1.0 °C∙s^−1^) is required to prevent the precipitation of secondary phases during the cooling stage (see Figure 5). Thus, the cast specimens were solubilized at 1075 °C for two hours and quenched in water. This solution heat treatment allowed a detailed analysis of the microstructure in the reinforced zone when subjected to a standard heat treatment cycle applied to austenitic stainless steel.

The microstructure of the heat-treated specimens was characterized by optical microscopy (OM) using a Leica DM4000 M with a DMC 2900 camera (Leica Microsystems, Wetzlar, Germany) and SEM/EDS. A detailed characterization of the phases was carried out by X-ray diffraction (XRD, Cu Kα radiation, Bruker D8 Discover, Billerica, Massachusetts, EUA), with a scanning range (2θ) of 5° to 100°, and by electron backscatter diffraction (EBSD). The EBSD data were subjected to a dilation clean-up procedure, using a tolerance angle of 15° and a minimum grain size of 10 points to avoid inaccurate predictions. Transmission electron microscopy (TEM) using a JEOL 2100 (JEOL Ltd., Tokyo, Japan) was also employed to characterize more exhaustively the phases that formed in the composite zone. In this sense, thin foils were prepared in a dual-beam focused ion beam (FIB) FEI Helios NanoLab 450S (FEI Company, Hillsboro, OR, USA) and fully identified through selected area electron diffraction (SAED) in TEM.

## 3. Results and Discussion

### 3.1. Characterization of the Initial Powders

**Figure 6:**
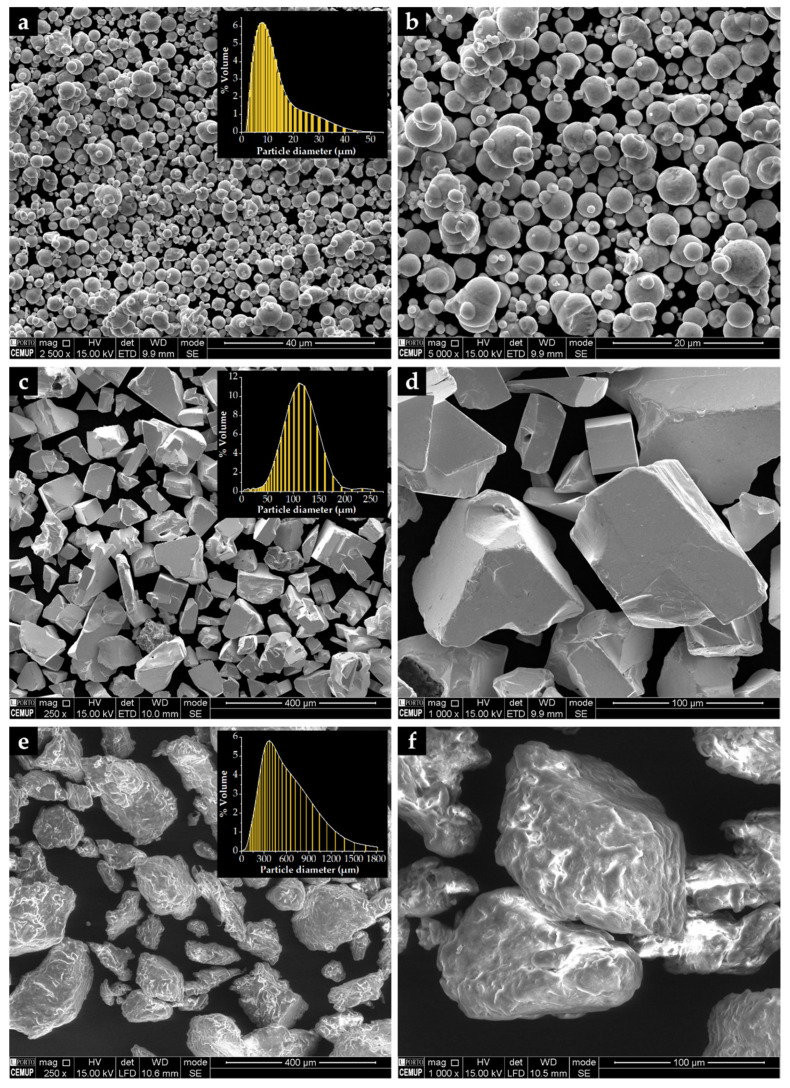
SEM-secondary electron (SE) images of the starting powders: (**a**,**b**) Fe with round morphology, (**c**,**d**) polyhedral particles of WC, and (**e**,**f**) M0101 binder with irregular shapes.

The characterization of the starting powders was the first step taken to evaluate the morphology and size distribution. The Fe powders are almost round, with an average diameter of 10 µm and a D_50_ of 8 µm (Figure 6a,b), unlike the WC powders that show a polyhedral shape (Figure 6c,d). These particles exhibit an average size of 106 µm and a D_50_ of 107 µm. The shape of M0101 binder powders is irregular, showing elongated and round particles (Figure 6e,f) with an average size of 475 µm and a D_50_ of 398 µm.

### 3.2. Characterization of the Porous Sintered Fe–WC Inserts

As mentioned above, parallelepipedal sintered inserts with dimensions of 31 mm × 12 mm × 9 mm were produced. It should be noted that the dimensions of the inserts were almost unchanged with the sintering.

**Figure 7:**
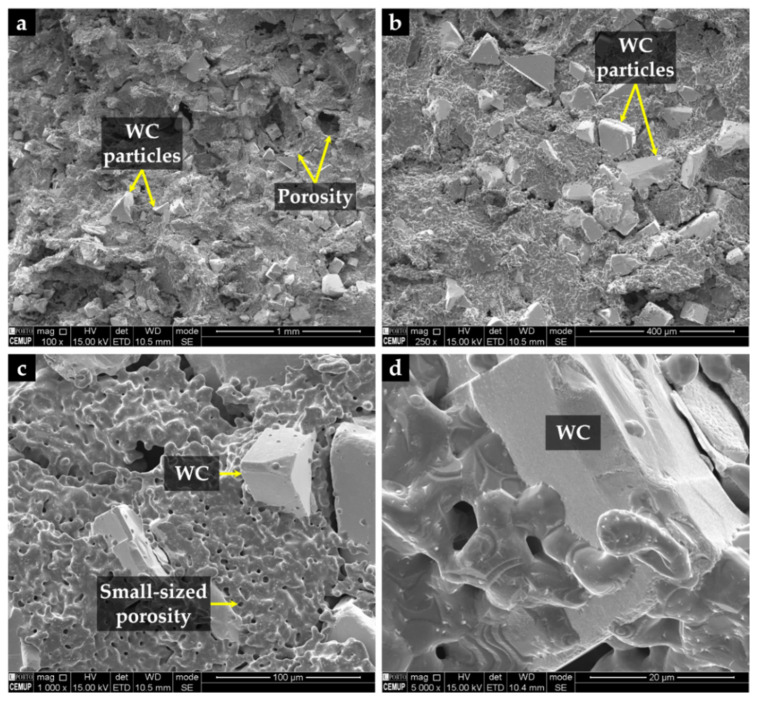
SEM-SE images of the surface of the porous sintered Fe–WC inserts: (**a**) Fe matrix with large WC particles and random porosity, (**b**) large polygonal-shaped WC particles with higher magnification, (**c**) small-sized porosity, and (**d**) wetting of a large WC particle by the iron.

It can be seen from Figure 7 that WC particles are well distributed in the Fe matrix, and Figure 7d highlights the suitable wettability of WC particles by the iron. As expected, cavities left by the binder removed during the debinding process were found, and a random distribution of larger (≥100 µm) and smaller porosities (≤10 µm) was observed.

### 3.3. Characterization of the Reinforced Specimens

**Figure 8:**
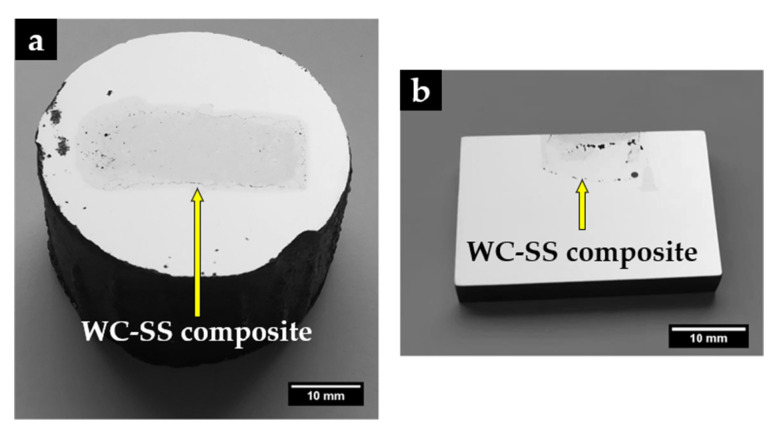
The reinforced specimen (**a**) and a cut through the cross-section (**b**), showing the depth of the composite zone.

Figure 8 shows a reinforced specimen with a polished surface in which it is possible to distinguish two zones: the composite zone (gray) and the base metal (light gray). As can be seen, the insert did not maintain the initial dimensions, indicating an extensive reaction of the sintered WC–Fe insert with the liquid metal. The composite zone has a length and width of 34 mm and 13 mm, respectively, and a regular depth of 6.5 mm. In the cross-section cut (Figure 8b), it is also possible to observe the presence of some defects in the composite zone, such as voids and a few cracks, that may have resulted from differences in the thermal expansion coefficients of the phases that constitute this zone (4.5–7.1 × 10^−6^ °C^−1^ for WC and 15–18 × 10^−6^ °C^−1^ for stainless steel), and from the fast cooling on heat treatment [4]. Nevertheless, the interface area is almost defect-free, as will be detailed throughout this study.

#### 3.3.1. Base Metal

**Figure 9:**
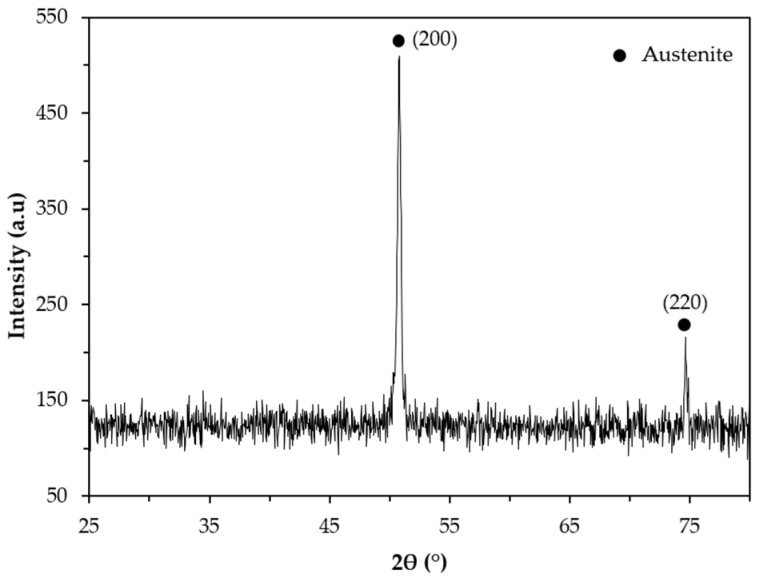
X-ray diffraction (XRD) pattern of the base metal in the 2θ range of 25°–80°.

**Figure 10:**
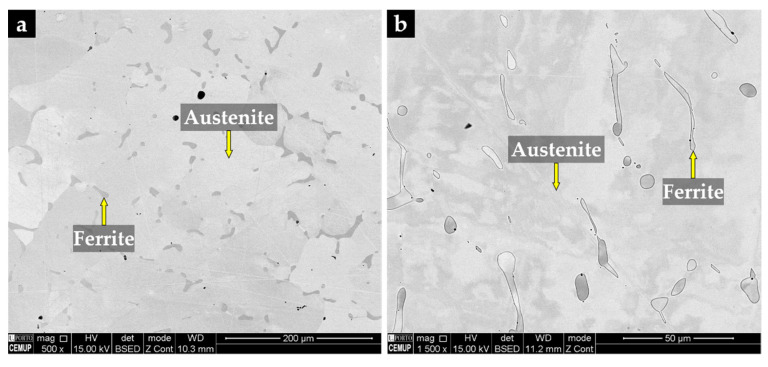
SEM image of the austenitic stainless steel after solution heat treatment at 1075 °C/2 h (**a**) and the same after chemical etching with 10% oxalic acid and higher magnification (**b**).

**Figure 11:**
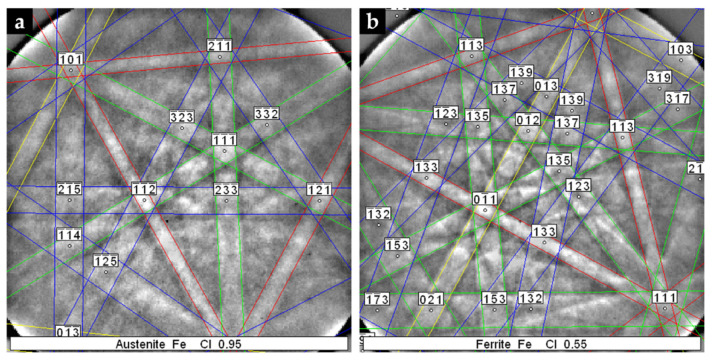
Indexed EBSD patterns corresponding to the phases that compose the microstructure of the austenitic stainless steel: austenite (**a**) and ferrite (**b**).

**Figure 12:**
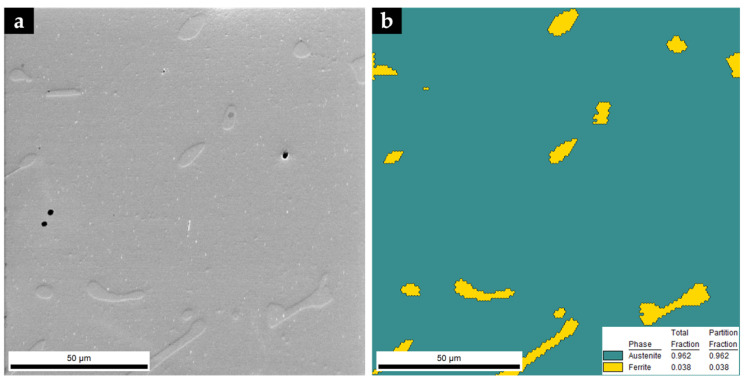
SEM image of the microstructure of the base metal (**a**) and EBSD phase map: γ in dark cyan and α in yellow (**b**).

The XRD pattern of the base metal presented in Figure 9 indicates the presence of austenite (γ). Nevertheless, SEM images of Figure 10 show pools of ferrite that were identified by indexed Kikuchi patterns and EBSD phase maps presented in Figure 11 and Figure 12, respectively. From the EBSD phase map of Figure 12b, we estimated a percentage of 4% of ferrite.

#### 3.3.2. Metal Matrix Composite Zone

**Figure 13:**
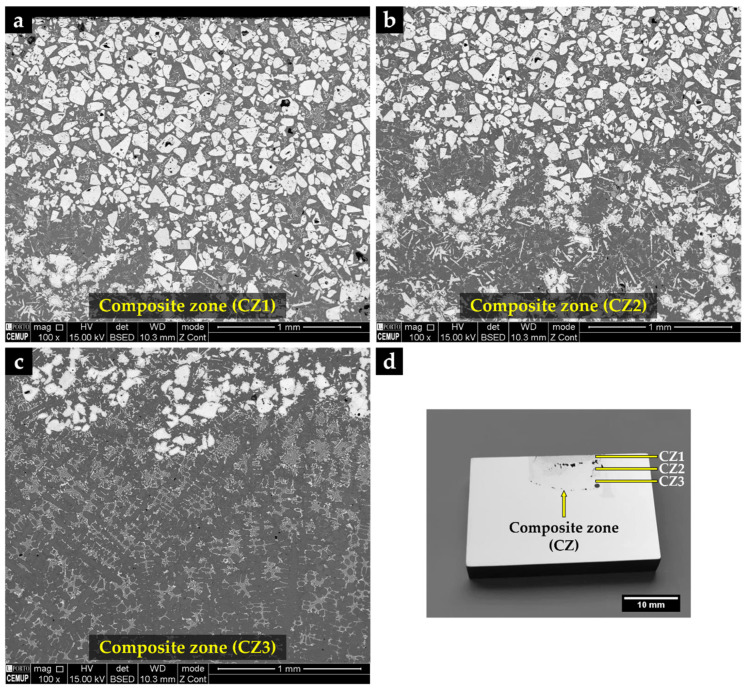
SEM-BSE images of the microstructure of the reinforced specimen showing three zones: composite zone nearest to the surface—CZ1 (**a**), intermediate composite zone—CZ2 (**b**), composite zone next to the base metal—CZ3 (**c**), and a cross-section of the specimen with the identified zones (**d**).

The metal matrix composite (MMC) shows three distinct zones, identified by CZ1 (zone nearest surface), CZ2, and CZ3 (inner zone near to the base metal), as can be seen in SEM images of Figure 13.

**Figure 14:**
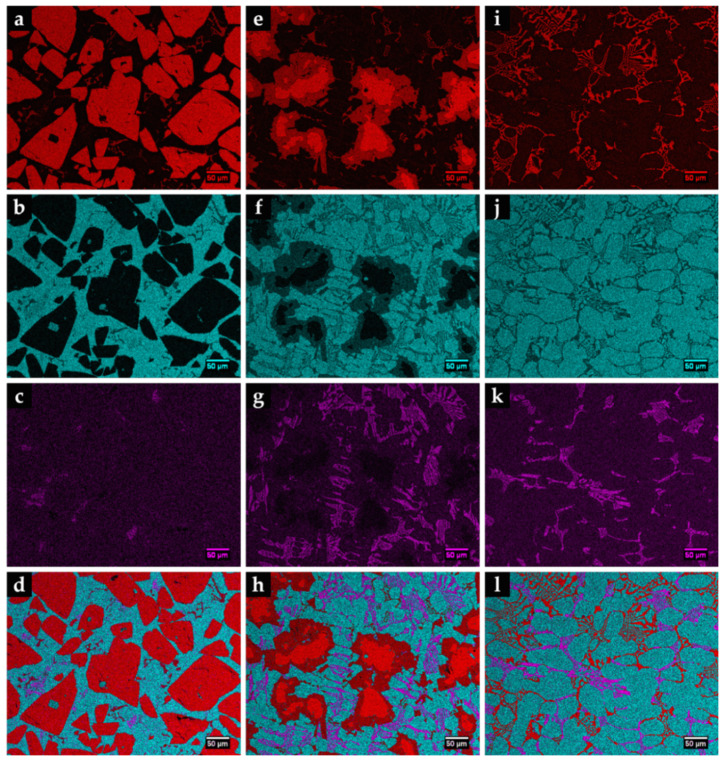
EDS elemental mapping of Fe (blue), Cr (pink), and W (red) for the microstructure of the three composite zones: CZ1 (**a**–**d**), CZ2 (**e**–**h**), and CZ3 (**i**–**l**).

The CZ1 and CZ2 zones are characterized by a high density of large polygonal-shaped white particles uniformly distributed (see Figure 13a). The EDS elemental maps, depicted in Figure 14, confirmed a W-rich phase, suggesting W carbides (Figure 14a,e). Fine light gray particles with plate shape also formed in CZ1. These particles contain W, Cr, and Fe, as shown in Figure 14a–c. A number of these light gray particles are also observed in the CZ2 zone, as displayed in Figure 13b. The CZ3 zone is characterized by a double-phase network in the interdendritic spaces (Figure 13c), rich in Fe, W, and Cr, as can be seen in Figure 14i–k.

**Figure 15:**
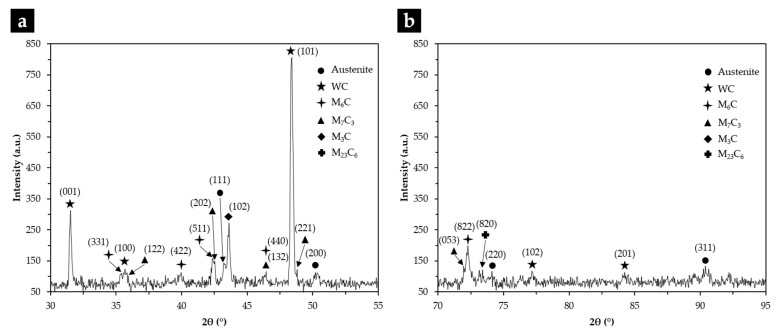
XRD patterns of the composite zone in the 2θ range of 30–55° (**a**), and 70–95° (**b**).

**Figure 16:**
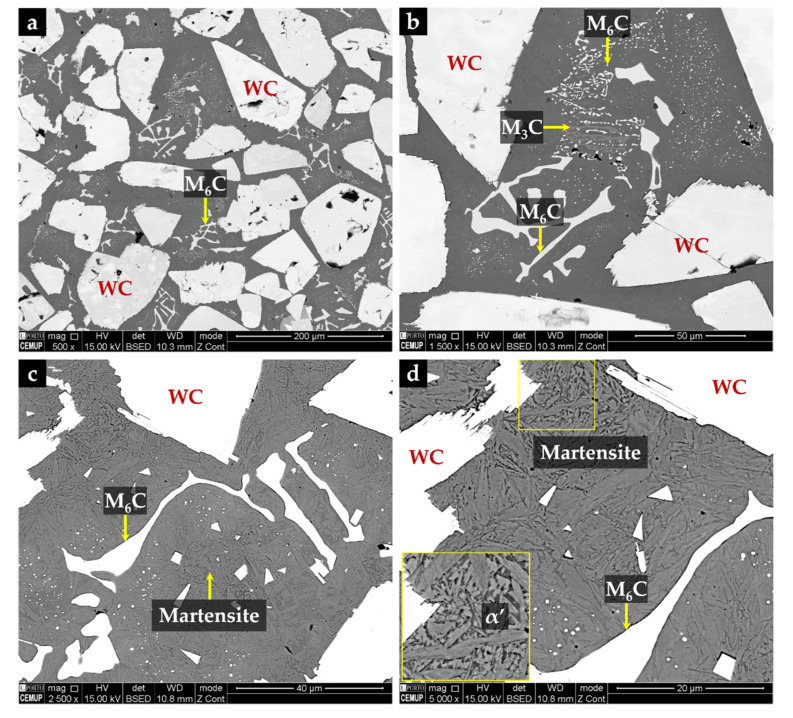
SEM-BSE images of the microstructure of the CZ1 composite zone: (**a**) uniform distribution of large polygonal-shaped WC particles and small particles of (Fe,W,Cr)_6_C, (**b**) higher magnification of (**a**) showing fine precipitation of (Fe,W,Cr)_3_C and (Fe,W,Cr)_6_C, (**c**) α’ matrix revealed with 10% oxalic acid, and (**d**) higher magnification of (**c**) showing the α’ plate-like morphology.

From the XRD results (see Figure 15) and the EDS elemental maps, we identified the fishbone precipitates as (Fe,W,Cr)_6_C and the fine plate-like precipitates as (Fe,W,Cr)_3_C, both observed in the CZ1 zone (Figure 16). The formation of these carbides can be explained by partial dissolution of WC particles in the liquid metal, leading to the precipitation at subsequent cooling [11,13]. Hackenberg et al. [41], found that found that the (Fe,W,Cr)_6_C carbides with fishbone shape form at high temperatures from the liquid, while fine plate-like (Fe,W,Cr)_6_C carbides precipitate from austenite at lower temperatures. For example, (Fe,W,Cr)_6_C carbide was found in austenitic stainless steels containing Mo, W, and Nb, after aging in the temperature range of 600–800 °C [41,42,43,44]. Lin [45] also found the (Fe,W,Cr)_3_C carbide in WC-304L stainless steel matrix composite produced by laser alloying.

**Figure 17:**
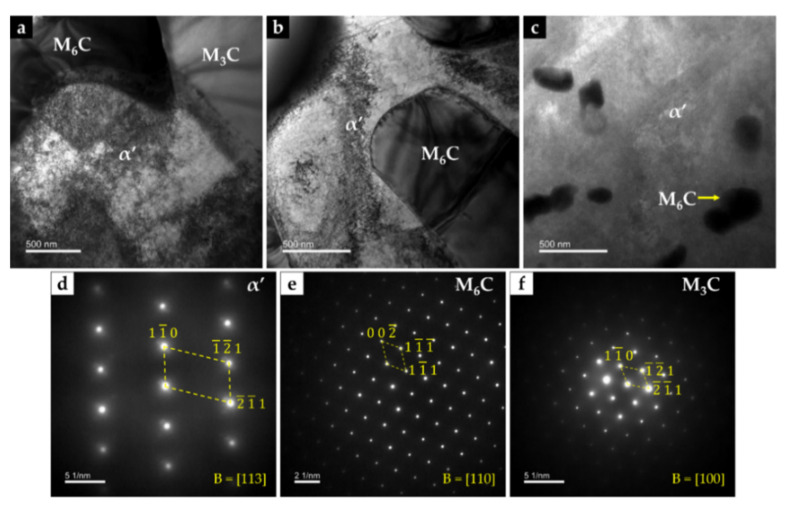
Dark-field TEM image of CZ1 composite zone: (**a**) interface between (Fe,W,Cr)_6_C, (Fe,W,Cr)_3_C, and α’, (**b**) (Fe,W,Cr)_6_C with a quasi-cuboid shape precipitated within α’ matrix, and (**c**) fine precipitates of (Fe,W,Cr)_6_C. SAED patterns were achieved by using the (**d**) [113] zone axis for α’, (**e**) [110] zone axis for (Fe,W,Cr)_6_C, and (**f**) [100] zone axis for (Fe,W,Cr)_3_C.

Chemical etching with 10% oxalic acid of the CZ1 zone revealed the presence of martensite (α’) in the matrix (see Figure 16c,d), confirming the destabilization of austenite, probably related to the precipitation of Cr-rich carbides that leads to reduction of the C and Cr content of the matrix [2,42]. TEM images of CZ1 zone, presented in Figure 17, give evidence of the martensite formation and (Fe,W,Cr)_6_C and (Fe,W,Cr)_3_C precipitation, as shown in Figure 17a. A coarse particle of (Fe,W,Cr)_6_C embedded in the α’ matrix can be observed in Figure 17b, and precipitation of small particles of (Fe,W,Cr)_6_C is shown in Figure 17c. Due to the presence of these phases, the average hardness of CZ1 composite zone is 655 HV 2 (four times harder than the base metal).

**Figure 18:**
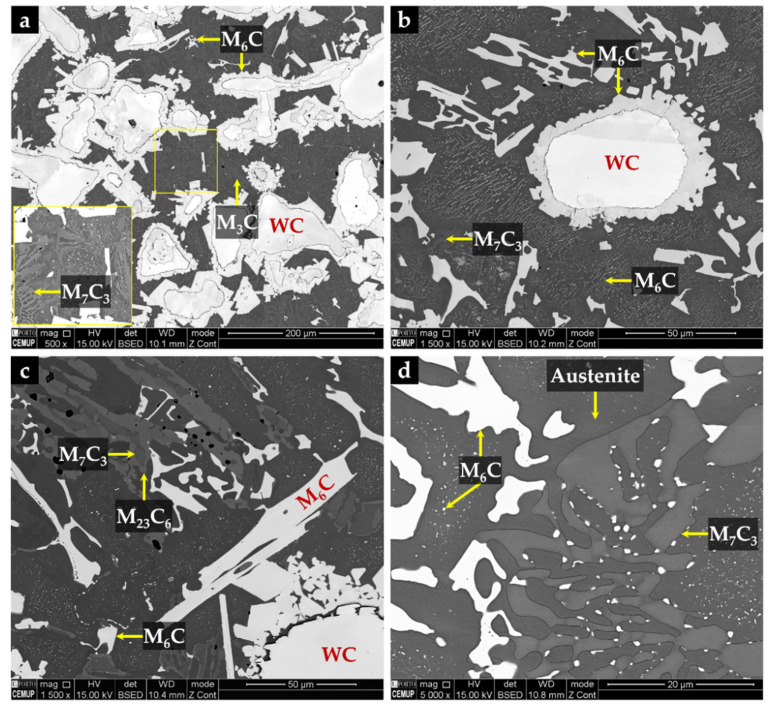
SEM-BSE images of the microstructure of CZ2 composite zone: (**a**) (Fe,W,Cr)_6_C precipitated around WC particles, (**b**) higher magnification of (**a**) showing fine plate-like precipitation of (Fe,W,Cr)_6_C, (**c**) carbides with a core of (Fe,Cr,W)_7_C_3_ and a shell of (Fe,Cr,W)_23_C_6_, and (**d**) higher magnification of (**c**) showing the multi-phase interdendritic network.

**Figure 19:**
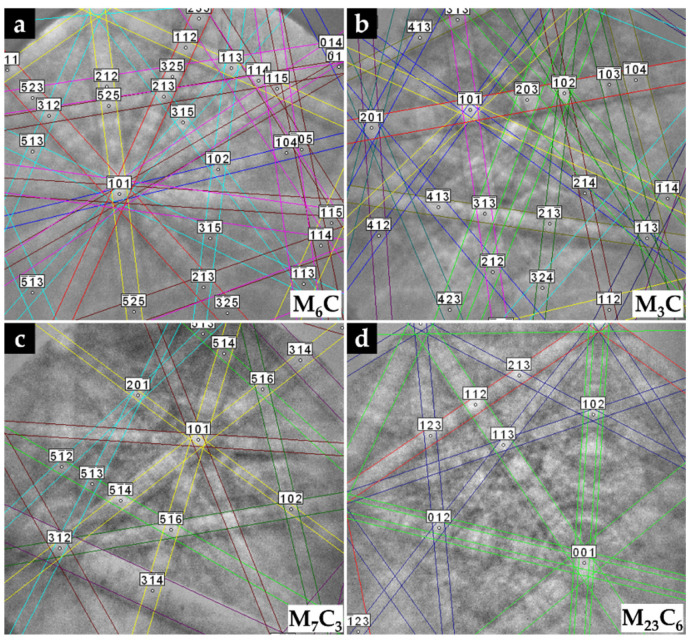
Indexed EBSD patterns of the carbides in CZ2 composite zone: (Fe,W,Cr)_6_C (**a**), (Fe,W,Cr)_3_C (**b**), (Fe,Cr,W)_7_C_3_ (**c**), and (Fe,Cr,W)_23_C_6_ (**d**).

The microstructure of the CZ2 zone shown in Figure 18 indicates that a strong dissolution of WC particles occurred with a consequent local enrichment of the matrix in C and W that, in turn, promoted the precipitation of (Fe,W,Cr)_6_C during slow cooling. Figure 18a–c show the precipitation of (Fe,W,Cr)_6_C around WC particles with an irregular form. The (Fe,W,Cr)_3_C were also detected in the CZ2 zone. Figure 19 provides the indexed Kikuchi patterns of the carbides formed in the CZ2 zone: (Fe,W,Cr)_6_C, (Fe,W,Cr)_3_C, (Fe,Cr,W)_7_C_3_, and (Fe,Cr,W)_23_C_6_. The (Fe,Cr,W)_7_C_3_ and (Fe,Cr,W)_23_C_6_ carbides were also identified from the XRD results, present in Figure 15. In Figure 18c, it is possible to observe carbides with a core–shell structure with a core of (Fe,Cr,W)_7_C_3_ and a shell of (Fe,Cr,W)_23_C_6_, suggesting the transformation of (Fe,Cr,W)_7_C_3_ into (Fe,Cr,W)_23_C_6_. A similar transformation was found in a cast austenitic stainless steel when exposed to the in-service temperature of 750–1000 °C [46].

**Figure 20:**
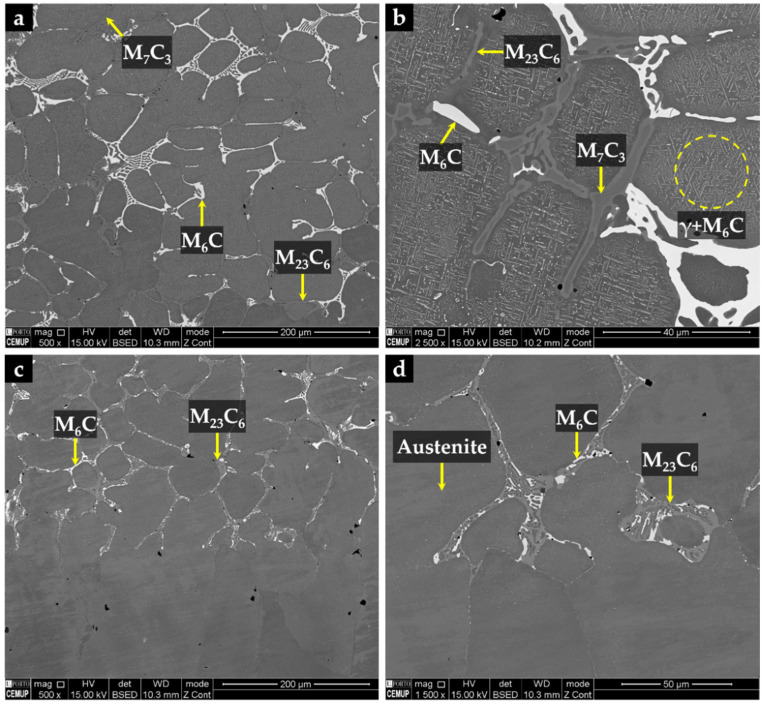
SEM-BSE images of the microstructure of CZ3 composite zone: (**a**) multi-phase interdendritic network of (Fe,Cr,W)_7_C_3_, (Fe,Cr,W)_23_C_6_, and (Fe,W,Cr)_6_C, (**b**) higher magnification of (**a**) showing fine precipitation of (Fe,W,Cr)_6_C inside the γ matrix, (**c**) bonding interface free of discontinuities, and (**d**) higher magnification of (**c**) showing the interdendritic precipitation at the bonding interface.

**Figure 21:**
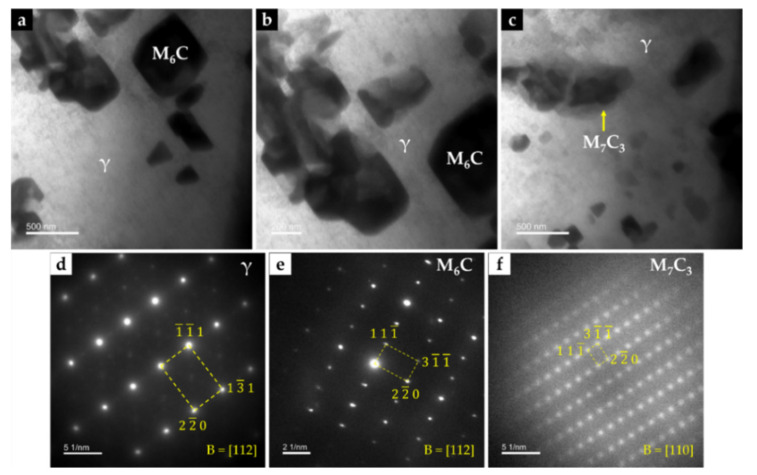
Bright-field TEM image of the CZ3 composite zone: (**a**) (Fe,W,Cr)_6_C with a cuboid shape precipitated within γ matrix, (**b**) higher magnification of (**a**), showing an agglomerate of (Fe,W,Cr)_6_C carbides, and (**c**) (Fe,Cr,W)_7_C_3_ precipitated within γ matrix. SAED patterns were achieved by using the (d) [112] zone axis for γ, (**e**) [112] zone axis for (Fe,W,Cr)_6_C, and (f) [110] zone axis for (Fe,Cr,W)_7_C_3_.

The CZ3 zone, next to the base metal, is characterized by coarse dendrites of austenite surrounded by a multi-phase interdendritic network of (Fe,Cr,W)_7_C_3_, (Fe,Cr,W)_23_C_6_, and (Fe,W,Cr)_6_C, as can be seen in Figure 20. Fine plate-like (Fe,W,Cr)_6_C carbides also precipitated in austenite dendrites, as shown in Figure 20b, and in more detail in TEM images of Figure 21. The microstructure of this zone also shows a bonding interface free of discontinuities and voids, indicating suitable wettability of the porous sintered WC–Fe insert by the liquid metal.

## 4. Conclusions

We successfully produced austenitic stainless steel specimens locally reinforced with WC–Fe inserts by employing an ex situ technique based on powder metallurgy technology. The defect-free interface between the composite zone and the base metal suggests that sound bonding was obtained with the methodology applied.

The produced reinforcement is essentially a graded material with a compositional and microstructural gradient along with its thickness. The microstructure of the zone closest to the surface is characterized by large polygonal-shaped WC particles and (Fe,W,Cr)_6_C and (Fe,W,Cr)_3_C carbides, with fishbone and plate-like shapes, respectively, that are formed from the reaction between the molten metal and the porous WC–Fe insert. The precipitation of these carbides led to C and Cr content reduction of the austenite, resulting in its destabilization and consequent transformation into martensite during cooling. In the inner zones, a strong dissolution of WC particles occurred, resulting in the precipitation of (Fe,W,Cr)_6_C carbides mostly around the WC particles. Additionally, the multi-phase interdendritic network composed of (Fe,Cr,W)_7_C_3_, (Fe,Cr,W)_23_C_6_, and (Fe,W,Cr)_6_C carbides that formed in this region disappears as we approach the matrix.

The sound interface bonding between the reinforcement and the base metal renders this approach, using the ex situ technique with WC particles, well suited for industrial applications.

## Figures and Tables

**Table 1:** Nominal chemical composition of the prepared austenitic stainless steel (wt %).

C	Si	Mn	P	S	Cr	Mo	Ni	Cu	N
0.06	0.79	0.80	0.03	0.01	18.30	2.41	11.07	0.31	0.06
